# 835. Biodegradable Temporizing Matrix: Infection Risk Factors and Management Strategy

**DOI:** 10.1093/jbcr/irag033.274

**Published:** 2026-04-06

**Authors:** Evie Medley, Julia Isber, Grayson Hetherington, David M Hill, Mahnoush Momeni, Matthew Sanders, Xiangxia Liu, Mahmoud Hassouba, Dani Kruchevsky

**Affiliations:** Firefighters Regional One Burn Center, Memphis, TN; Firefighters Regional One Burn Center, Memphis, TN; Firefighters Regional One Burn Center, Memphis, TN; Firefighters Regional One Burn Center, Memphis, TN; University of Tennessee, Memphis, TN; Firefighters Regional One Burn Center, Memphis, TN; Firefighters Regional One Burn Center, Memphis, TN; Firefighters Regional One Burn Center, Memphis, TN; Firefighter's Regional One Burn Center, Germantown, TN

## Abstract

**Introduction:**

Over the past decade, synthetic dermal substitutes have reshaped the reconstructive ladder by offering alternatives that facilitate wound closure and improve graft take. Among these, biodegradable temporizing matrix (BTM) is well established for its ability to resist infection and generate a durable neodermis. Nonetheless, BTM remains susceptible to infections that may compromise healing, precipitate sepsis, necessitate removal of the matrix, and increase morbidity. With proper response, these infections can be mitigated, and the matrix can be salvaged. The aim of this study is to identify risk factors associated with BTM infection and to evaluate the effectiveness of treatment strategies in managing these cases.

**Methods:**

A retrospective chart review was performed at a level 1 trauma and American Burn Association (ABA)-verified burn center for patients 18 years and older who underwent BTM placement between April 2022 and April 2025.

**Results:**

In our cohort, 65 patients underwent wound reconstruction with BTM. Placement on the torso and use in burn injuries were associated with a higher risk of local infection. The management approach consisted of local infection control through irrigation beneath the silicone layer and application of sodium hypochlorite dressings. Early partial delamination was required only in rare cases (4/65, 6.2%). With this approach, BTM salvage was achieved in all cases, and no patient required acute surgical excision of the dermal substitute.

**Conclusions:**

Our study demonstrated predisposing factors for clinical infection of BTM that may assist clinicians with early diagnosis and treatment. Moreover, we present a salvage strategy that can be applied in cases of infection, allowing majority of patients to achieve successful integration of the BTM.

**Applicability of Research to Practice:**

Results provide practical guidance for clinicians on risk stratification and infection control in BTM use.

**Funding for the study:**

N/A.

